# Erratic Behavioral Attitude Leads to Noncommunicable Diseases: A Cross-Sectional Study

**DOI:** 10.1155/2020/7257052

**Published:** 2020-01-10

**Authors:** Arif Habib, Mohammad Mahtab Alam, Izhar Hussain, Nazim Nasir, Musa Almuthebi

**Affiliations:** ^1^Department of Public Health, College of Applied Medical Sciences Khamish Mushait, King Khalid University, Abha, Saudi Arabia; ^2^Department of Basic Medical Sciences, College of Applied Medical Sciences, King Khalid University, Abha, Saudi Arabia; ^3^Department of Anesthesia, College of Applied Medical Sciences, King Khalid University, Abha, Saudi Arabia

## Abstract

**Background:**

The burden of noncommunicable diseases (NCDs) in the Arab world has reached an alarming level. Behavioral risk factors including consumption of fast food, inadequate sleep, and skipping meals are pervasive. This study aims at establishing the association between NCDs and modernized behavioral risk factors among populations.

**Methods:**

A cross-sectional study was carried out with 1070 respondents who were 18 years old. The data were collected using a structured questionnaire with the help of the WHO STEPS approach with some modifications regarding NCD risk factors.

**Results:**

30% of respondents had cardiovascular diseases (CVD) followed by respiratory diseases (23%) and diabetes (3%) while the habit of smoking was found among 52% of respondents followed by physical inactivity (49%), skipping meals (24%), and inadequate sleep (30%). Consumption of fast food was found to be a significant risk factor for obesity (odds ratio (OR) = 2.72, 95% confidence interval (CI) [1.50, 4.92]), CVD (OR = 1.52, 95% CI [1.20, 1.94]), and respiratory disease (OR = 2.13, 95% CI [1.58, 2.86]). Significant linkages were found between CVD and smoking (OR = 0.69, 95% CI [0.54, 0.88]), diet pattern (OR = 1.86, 95% CI [1.44, 2.39]), fast food (OR = 1.52, 95% CI [1.20, 1.94]), and sleep hours (OR = 0.57, 95% CI [0.42, 0.79]).

**Conclusions:**

Undesirable behavioral risk factors pose a considerable threat to public health with a high prevalence rate of NCDs. Reducing the NCD burden and promoting healthy lifestyle formation of suitable strategies and their smooth implementation is the need of the hour.

## 1. Introduction

With the epidemiological transition, noncommunicable diseases (NCDs) are a major concern all over the globe. Their prevalence has been found to be around 40% in developing countries and 10% in developed countries, in the age group of 30–70 years. NCDs are also responsible for high economic burden on a country by affecting the productivity of its citizenry and high treatment cost as well. Patients affected by lifestyle-induced NCDs have the highest rate of premature mortality. The prime cause of high magnitude of NCDs is unhealthy dietary behavior of the population concerned.

In earlier times, communicable diseases were the major cause of deaths, but now NCDs have replaced them as the major causative agents of mortality. Data show that 57% of annual deaths were caused by CVDs, diabetes, chronic respiratory diseases (CRDs), and cancers in the majority of the countries in the Eastern Mediterranean Region (EMR) in 2012 [1].

According to a recent WHO report, NCDs cause 41 million deaths every year, which is nearly 71% of total deaths across the globe. NCDs kill around 15 million people each year who are under the age group of 30 to 69 years and over 85% of these deaths take place in developing countries [2].

According to the report by the WHO (2014), NCDs are estimated to account for 78% of total deaths, out of which CVDs claimed maximum lives (46%) followed by cancers (10%), diabetes (5%), COPDs (3%), and other NCDs (14%) [3].

Treatment for an NCD is a costly affair. It is estimated that diabetes treatment costs SAR 17 billion ($4.5 billion) while the treatment for cancer is estimated at SAR 82,500 ($22,000) per patient per year, and it is reported that by 2030, the financial burden of cancer may exceed SAR 2 billion per year. In 2015, the treatment for asthma aggregated SAR 10 billion, and it is estimated that by 2030, the treatment of asthma may exceed SAR 20 billion [4].

Other than unhealthy dietary behavior, some economic and social crises are also linked to NCDs. High rate of unemployment, unhealthy work environment, low wages, and high workload were also held responsible for an increased rate of mood disorders, anxiety, depression, and suicide. Studies indicated that these problems may have also affected the general health of workers by increasing the risk of cardiovascular and respiratory diseases [5, 6].

In 2016, the population of Saudi Arabia was over 31 million individuals, all of whom require healthcare services in some capacity. In 2016, children and youth under 15 years of age represented 30.35% of the Saudi population, adults between 15 and 64 years of age represented 65.46%, whereas seniors over the age of 65 comprised 4.17% of the population. In 2016, Ministry of Health's Statistical Yearbook reported the total number of visits to the primary healthcare centers and private, general, and polyclinics was nearly 138 million visits; 3,451,377 cases represented the total number of inpatients in the hospitals of all health sectors: 49.4% of them were in MOH hospitals. The statistical yearbook also reported 49,817,811 visits to health centers and 64,346,910 visits by outpatients to MOH hospitals.

The alarming burden of NCDs is due to the rising ubiquity of behavioral and metabolic risk factors for these noncommunicable diseases [7]. A risk factor is an attribute, or subjection of a person, which increases the odds of contracting a noncommunicable disease. Knowledge about these risk factors can be used to reduce the distribution of these risk factors considerably. The risk factors are classified into 3 types: behavioral, metabolic, and biochemical. Behavioral risk factors include smoking, alcohol consumption, unhealthy diet intake, low fruit and vegetable consumption, stress, and sedentary lifestyle. However, there are other risk factors such as irregular meal patterns, fast food consumption, and inadequate sleep. These are equally responsible for the development of NCDs as established risk factors in a population. The study was conducted with the objectives to examine these risk factors jointly with NCDs, especially in the Arab world. This study describes the association of NCDs with social and individual behavior.

## 2. Materials and Methods

### 2.1. Type of Study

This is a cross-sectional epidemiological study on NCD risk factors applying a modified WHO STEP-1 procedure in the capital city of Asir Province near the Red Sea in southwest Saudi Arabia. This article aims at describing the behavioral risk factors of NCDs among the population residing in Abha area and determining the association between behavioral risk factors.

### 2.2. Population under Study

The population of the city is about half a million. In this study, we consider the population more than or equal to 18 years of age visiting primary healthcare centers at any time for any purpose.

### 2.3. Sample Population

During the literature review, we did not find any study which has reported the prevalence of the residents visiting PHCs of their area. We conducted a small survey by selecting 100 persons randomly and found that 27 individuals from the age of 18 to 71 years visited PHCs. Taking prevalence of 27% with a margin of error of 0.10% and the level of significance 5%, we include 1070 samples in our study.

### 2.4. Inclusion Criteria

The inclusion criterion for the study was that of an adult population of age more than or equal to 18 years visiting PHC in their respective areas for any purpose and apparently healthy and willing to participate in the study.

### 2.5. Exclusion Criteria

The exclusion criteria for the study were that of persons less than 18 years of age, females who were pregnant, psychiatric patients, severely ill, and those who rejected to participate in the study.

### 2.6. Sampling Procedure

In Abha district, the total number of PHCs is 46 out of which 12 are in urban and 34 are in rural areas. The ratio of urban to rural is approximately 1 : 3. We randomly selected 2 PHCs from urban and 6 PHCs from rural areas by using lottery method, and then from each PHC, researcher selected samples as per the population ratio of each PHC using stratified sampling comprising of 1,070 subjects. The probability proportional to size sampling procedure has been adopted to meet the feasible acceptance of the results.

### 2.7. Allocations of Samples to Different Strata/Blocks

In the present study, we estimated the proportion by stratified sampling. We allocated the samples in different strata in each block as per proportional allocation. We took the average of the total number of registrations of patients of the weeks of the last five months of the year 2018.

### 2.8. Study Tool

A standardized pretested, structured questionnaire consists of the sociodemographic particulars and details regarding behavioral risk factors for noncommunicable disease.

### 2.9. Behavioral Risk Factors

 
*Tobacco* users were defined as individuals who had the habit of using any form of tobacco/shisha
 
*Physical activity:* low physical activity was defined as <150 minutes of moderate physical activity per week or no activity at all
 
*An unhealthy diet* is the low consumption of fruits and vegetables at less than five servings per day
 
*Fast food consumption* was defined as an individual consuming fast foods thrice or more per week
 
*Skipping meal* included individuals who had a habit of skipping meals routinely or at least 5 times per week
 
*Inadequate sleeping* included individuals sleeping less than 8 hours per day routinely

### 2.10. Data Collection

The study used a pretested structured questionnaire which was conducted by using the modified WHO NCD STEPS approach I instrument version 2.2 (WHO (2014)). The protocol was based on the WHO STEPS approach. Information on sociodemographic variables and behavioral risk factors, such as tobacco use, physical exercise, diet pattern, consumption of fast food, skipping routine meals, and inadequate sleep was collected by using a pro forma translated in Arabic language to ensure that all respondents understood each and every question.

The selected subjects were briefed about the study, and proper consent in a written format (enclosed with the questionnaire) was obtained from them.

All the questions which have been considered were adequately reliable with Cronbach's alpha of 0.767. The primary information was collected (May to September 2018) from 1070 participants who visited eight selected PHCs in two areas of district Abha: Asir, KSA.

### 2.11. Data Analysis

The responses were collected and counted manually based on the options specified for each question framed. Files on each participant (questionnaire) were then merged using the participant identity number crosschecked with participant name and identification number. After merging, common variables in the dataset were matched and inconsistencies were corrected. All the data collected were entered into Microsoft Excel. Data cleaning and analysis was done using SPSS 25 version. The prevalence of NCD risk factors has been presented in the form of frequencies and percentages. Most of the variables in this study are categorical. Statistical significance (chi-square test and *P* value) and strength of association (odds ratio and 95% confidence interval) were tested between behavioral risk factors.

## 3. Results

A total of 1070 subjects took part in the study. The population consists of rural (*n*=677) and urban patients (*n*=393) visiting PHCs. The female and male percentages were found to be 36% and 64% and 41% and 59% among rural and urban subjects, respectively. It denotes the greater participation of males from rural as well as urban areas and thus the utilization of PHC services as well.

The highest number of patients was found in the age group of 26 to 40 years (42% (*n*=291) and 57% (*n*=224) in the rural and urban subjects, respectively), while the least number of patients was found in the age group of more than 60 years (13% (*n*=86) and 6% (*n*=24) in the rural and urban areas, respectively). It shows that old age people who are more than 60 years are availing least services of PHCs in comparison with the younger people. The reason might be the lack of mobility and self-dependence among older people.

Among rural subjects, the maximum number of patients (*n*=252, 37%) was educated up to secondary level, while 12% (*n*=83) were found to be not literate at all. On the other hand, maximum number of patients (*n*=246, 63%) from the urban area was university graduates, and only 2% (*n*=8) were found to be not eligible to read and write at all. It denotes the higher literacy rate among urban subjects than rural patients.

The maximum number of subjects in a rural area (*n*=353, 52%) was married while 3% (*n*=21) were divorced. Similarly, 59% (*n*=230) were married in an urban area while 10% (*n*=38) were found to be divorced.

The maximum (*n*=255, 38%) were unemployed in a rural area while 37% (*n*=251) were having income less than 7000 SAR/month. Among urban subjects, the maximum (*n*=156, 40%) had income between 7000 and 15000 SAR/month and 26% (*n*=104) were unemployed. It denotes the better economic condition among urban subjects which might be the reason for higher health-seeking behavior (Table 1).

Maximum number of patients (*n*=202, 30) had complaints of cardiovascular diseases (CVD), which include stroke, angina, coronary artery diseases, and so on followed by respiratory diseases (*n*=156, 23%). Diabetes and cancer had been reported by (*n*=21) 3% and (*n*=2) 0.29%, respectively. Among urban subjects, maximum number of patients (*n*=158, 40%) had a complaint of CVD followed by respiratory diseases (*n*=59, 15%) while diabetes and cancer had been reported by (*n*=27) 7% and (*n*=9) 2% of subjects. It shows the sizeable prevalence of CVD among rural as well as urban populations followed by respiratory diseases, diabetes, and cancer with a greater share among the urban population. It also warrants the designing and implementation of the strategies for prevention, early diagnosis, and better management of noncommunicable diseases among rural as well as urban population (Figure 1).

Smoking habit was more prevalent among urban patients (*n*=214, 54%) in comparison with their rural counterparts (*n*=324, 48%). Majority of rural patients were found to be physically inactive as (*n*=278) 40% of patients accepted to exercise occasionally while urban subjects were found to be the most active as (*n*=113) 29% accepted to exercise daily. Majority of patients from rural (*n*=425, 63%) as well as the urban areas (*n*=350, 89%) accepted to have food at home. Majority of subjects from both the areas accepted to have 3 meals a day. Most of the rural (*n*=418, 62%) and urban subjects (*n*=268, 68%) reported to have a fondness for fast food. Majority of rural (*n*=653, 96%) as well as urban subjects (*n*=282, 72%) accepted to have a sleep pattern of less than 8 hours daily, which is not a good sign in terms of a healthy lifestyle. 35% (*n*=240) and 34% (*n*=228) of rural subjects reported taking canned juice and soft drinks, respectively, while 38% (*n*=150) and 37% (*n*=147) of urban subjects accepted taking fresh juice and soft drinks daily (Table 2).

The magnitude of various behavioral risk factors was analysed and is represented in odds ratios and confidence intervals. Further, the linkage between common risk factors versus pathophysiological risk factors such as obesity, cardiovascular disease, and respiratory disorders was established in order to find independent associations. Consumption of fast food was a significant risk factor for all three conditions: odds ratio (OR)  = 2.72 and 95% CI [1.50, 4.92] for obesity, OR = 1.52 and 95% CI [1.20, 1.94] for CVD, and OR = 2.13 and 95% CI [1.58, 2.86] for respiratory disease. There were no significant associations between sleep hours and obesity. However, obesity was significantly associated with consumption of fast food (OR = 2.72, 95% CI [1.50, 4.92]) and diet pattern (OR = 4.34, 95% CI [2.19, 8.60]). Smoking (OR = 0.76, 95% CI [0.42, 1.37]) had opposite effect on obesity compared to nonsmokers (OR = 2.78, 95% CI [0.98, 7.82]).

Table 3 shows multivariate analysis of risk factors associated with CVD. There were significant associations between CVD and smoking (OR = 0.69, 95% CI [0.54, 0.88]). This habit is more dangerous as it damages the lining of the smoker's arteries. Diet pattern (OR = 1.86, 95% CI [1.44, 2.39]) and fast food (OR = 1.52, 95% CI [1.20, 1.94]) have also good impact on developing CVD. We did not find any association between sleeping hours and CVD (OR = 0.57, 95% CI [0.42, 0.79]).

Table 4 shows behavioral analysis risk factors associated with RD.

RD showed significant associations with smoking (OR = 0.22, 95% CI [0.15, 0.32]) and less physical activity (OR = 0.53, 95% CI [0.38, 0.73]). Fast food consumption and more sleeping hours are significantly associated with RD ((OR = 2.13, 95% CI [1.58, 2.86]) and (OR = 0.33, 95% CI [0.23, 0.46]), respectively) (Tables 3–5).

## 4. Discussion

The Saudi population is growing rapidly due to the high birth rate/life expectancy and low infant mortality rate [8]. Saudi population will be 39.8 million in 2025. This high population growth has demanding feedback on healthcare services and facilities. The Saudi community has been positively affected in their income [9], and the country was ranked 0.75 in Human Development Index, becoming 55th in 194 countries, which has led to better services that included healthcare. Approximately 80% of the Saudi health services are government-funded and provided free of charge to the whole population [10].

NCD is the largest contributor to the disease burden, and its prevalence is expected to continue to rise [3]. It is because of the consequence of the changing patterns of disease from communicable to noncommunicable diseases [11]. There is an alarming increase in cardiovascular, chronic respiratory diseases, diabetes, and cancer, and it accounts for 78% of all mortality [2, 6, 12].

Our study aims at providing a commencement of NCD risk indicators in Asir region. Overall cardiovascular disease has been highest among NCDs. Prevalence is higher in urban than in rural areas. On the other hand, respiratory disease is higher in rural than in urban areas. There is a sizeable deviation between the areas due to demographic and socioeconomic contrast. Also it has been observed that low wages and unemployment have a direct impact on the development of NCDs. There are various other studies which endorsed our observations. When comparing to an earlier study [6, 12, 13], we find evidence of an increase in some NCDs. The study indicates that the urban population is more prone to developing CVD and diabetes. The most prevalent factors are low physical activity, unhealthy dietary pattern, skipping meals, consumption of fast food, and inadequate sleeping. These factors are more common in the urban population. Respiratory disease is a very alarming sign in this area because of high altitude, wind movement, and changes in temperature.

Our findings confirm that smoking habits are slightly higher in urban than in rural areas. It is an established fact that smoking is the reason for growing CVD, respiratory disease, and other NCDs. In the kingdom, cigarette prices are higher than in other GCC countries. Since January 2017, the excise duty, commonly known as a “sin tax,” is imposed on unhealthy products. This work describes that there is no consequential decrease in the frequency of smoking even after the increase in tobacco rates. It is a well-established fact that tobacco use is the most common cause of noncommunicable diseases. By 2020, WHO assesses that tobacco will be the reason for 7.5 million deaths per annum or it approximates one in per ten mortality rates [2]. Smoking use is the common risk factor among the four main groups of NCDs—cardiovascular disease, chronic respiratory disease, diabetes, and cancer. It is also an established risk indicator of communicable diseases such as tuberculosis, bronchitis, and lower respiratory tract infections—health hazards that plague humanity.

Majority of the studied subjects (more than half) reported being physically inactive. Besides men and women, the young generation was more physically inactive. People rarely go for jogging or simply walk. Saudi Arabia's economic transition has changed conventional state and vocational customary behavior to the affliction of physical inactivity [6, 13].

The provision of diet prevailing in developed countries is characterized by high utilization of sifted grains, mutton, desserts, sweets, French fries, and high-fat dairy products [5, 14]. Harmful patterns of food intake have a direct strong relation with metabolic changes. This pattern is a significant reason for the incidence of noncommunicable diseases (NCDs). It has also been associated conclusively with an increased frequency of respiratory disease [2]. Further, a vital incitement with fast food meals has been reported to aggravate air-shaft inflammation. There are a number of research studies which reported that the “Western” diet is directly related to an increased risk of respiratory disease [5].

Skipping meals is also a problem in the population. It has been found that twenty-four percent in rural and thirty percent in the urban population are skipping at least one meal mostly breakfast per day. The main reason for skipping breakfast was related to inadequate sleep. We found that more frequent breakfast skipping was associated with greater odds of being overweight. This behavior was reported mostly in young people. This gives rise to the growth of NCDs among the population [12, 15]. Most of the studies endorse that cardiovascular risk factors are physical inactivity, smoking, high blood pressure, and high serum total cholesterol [16].

Consumption of processed fast food continued to increase swiftly in the kingdom. This sustenance adaptation affects dietary patterns and nutrition intake, which increase the likelihood of spreading NCDs. It has been found that the persons among the population who remain out most of the time from home incline to contemplate ease, accessible, and approach of eating as an important factor in fulfilling their food and energy requirements. This situation may lead to an imbalance in their health status and risk of diet-related noncommunicable diseases (NCDs) [17].

There is sufficient evidence of an association between fast food and diabetes [17]. It has been reported that the Saudi population shows affection for pastry desserts, creamed sandwiches, French fries, and fast foods. This behavior increases the risk factors for noncommunicable diseases among the population as there are a number of studies that reported the association between fast food consumption and increase in body mass index which results in obesity, cardiovascular disease, and so on. The other major challenging area of consumption of fast food is energy drinks and carbonated soft drinks that results in the production of immoderately refined sugar which can also lead to obesity and hence added to the risk factors of NCDs [14, 18].

Adequate sleeping is as important as a balanced diet and physical activity. It is incredibly essential for our health. Most of the experts declare that one should get at least seven hours of sleep each night. Unfortunately, the present behavioral environment intervenes in the natural sleep pattern. Most of the persons, particularly adult populations, are now sleeping less than they require. The quality of sleeping has been decreased which results in inefficient concentration towards work or physical activity and towards eating adequate meals. It has been reported that poor sleep leads to an increase in weight significantly more than those persons who get adequate sleep. There are many risk factors caused by this behavior which cause heart disease and stroke. It also affects glucose metabolism, depression, and type 2 diabetes. There is a strong direct relationship between inadequate sleep and the long-term inflammation of the digestive system. This behavior is a silent indicator for an increasing frequency of noncommunicable disease. Besides the intervention of established risk factors, the public health and educational institutes should lay stress on the importance of adequate sleep, health hazards of consumption of fast food, carbonated soft drinks, and skipping meals.

## 5. Conclusion

There is strong evidence of unusual lifestyle-related behaviors among the population. This is the reason for the increasing prevalence of noncommunicable diseases and subsequent unwanted economic burden. Prevention can happen by the use of every means available including media, counseling, and social activities, and such actions should be replicated to address the behavioral attitude among the population at global, regional, and national levels.

## Figures and Tables

**Figure 1:**
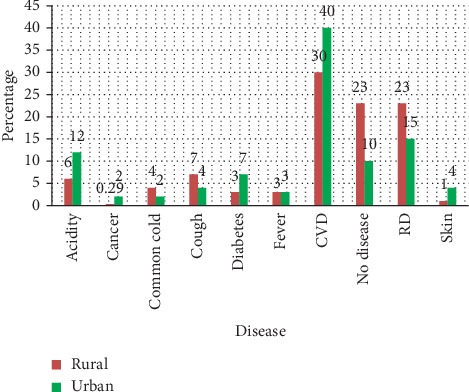
Prevalence of the disease among the population to visit PHCs.

**Table 1:** Sociodemographic characteristics of the studied population.

Parameter	Area				Total (*n*=1070)
	Rural (*n*1=677)	%	Urban (*n*2=393)	%	
Gender					
Female	245	36	160	41	405
Male	432	64	233	59	665
Age group					
<25	120	18	62	16	182
26–40	291	42	224	57	515
41–60	180	27	83	21	263
>61	86	13	24	6	110
Education level					
Illiterate	83	12	8	2	91
Primary	155	23	56	14	211
Middle	115	17	16	4	131
Secondary	252	37	67	17	319
University	72	11	246	63	318
Marital status					
Divorced	21	3	38	10	59
Married	353	52	230	59	583
Single	303	45	125	32	428
Monthly income					
<7000	251	37	133	34	384
7000–15000	171	25	156	40	327
No income	255	38	104	26	359
Employment status					
Employed	422	62	289	74	711
Not employed	255	38	104	26	359

**Table 2:** Behavioral attitude of the studied population in comparison with their respective areas.

Variables	Area				Total	Chi-square; significance
	Rural (*n*1=677)	%	Urban (*n*2=393)	%		
Smoking habit						
No	353	52	179	46	532	4.3258; *P* < 0.05
Yes	324	48	214	54	538	
Physical activity						
Daily	53	8	113	29	166	141.2; *P* < 0.05
Every alternate day	19	3	31	8	50	
Occasionally	278	41	56	14	334	
Do not exercise	327	48	193	49	520	
Routine meals						
Home	603	89	290	74	893	42.0457; *P* < 0.05
Restaurant	74	11	103	26	177	
How many times do you eat daily						
2 times	188	24	120	30	308	224.85; *P* < 0.05
3 times	309	45	177	45	486	
4 times	180	27	88	22	268	
More than 4	0	0	8	2	8	
Do you like fast food						
No	259	38	125	32	384	4.4966; *P* < 0.05
Yes	418	62	268	68	686	
Sleep daily						
=8 hours	24	4	111	28	135	165.83; *P* < 0.05
<8 hours	653	96	282	72	935	
What drink do you take daily						
Canned juices	240	35	72	18	312	87.1209; *P* < 0.05
Energy drink	96	14	24	6	120	
Fresh juices	113	17	150	38	263	
Soft drink	228	34	147	37	375	

**Table 3:** Association between behavioral risk factors and cardiovascular disease.

Risk factors	Behavior	Total participants (*n*=1070) (%)	CVD frequency (*n*=360) (%)	Odds ratio	95% CI	Chi-square value	*P* value
Smoking habit	Yes	538 (50.28)	213 (59.16)	0.6979	0.54–0.88	8.5301	0.003493
	No	532 (49.72)	147 (40.83)				
Physical activity	Yes	216 (20.18)	51 (14.16)	1.5324	1.09–2.13	6.42	0.01122
	No	854 (79.82)	309 (85.84)				
Diet pattern	Adequate	793 (74.11)	218 (60.55)	1.8648	1.44–2.39	23.89	0.0001
	Inadequate	277 (25.89)	142 (39.45)				
Routine meals	3 times	603 (56.35)	254 (70.55)	0.8490	0.67–1.06	22.61	0.001
	Skipping meals	467 (43.65)	106 (29.45)				
Consumption of fast food	Yes	686 (64.11)	194 (53.89)	1.5286	1.20–1.94	11.89	0.0005
	No	384 (35.89)	166 (46.11)				
Sleep hours	=8 hours	135 (12.61)	72 (20)	0.5775	0.42–0.79	11.86	0.0005
	<8 hours	935 (87.39)	288 (80)				

**Table 4:** Association between behavioral risk factors and respiratory disease.

Risk factors	Behavior	Total participants (*n*=1070) (%)	RD frequency (*n*=215) (%)	Odds ratio	95% CI	Chi-square value	*P* value
Smoking habit	Yes	538 (50.28)	176 (81.86)	0.2241	0.15–0.32	72.3132	0.0001
	No	532 (49.72)	39 (18.14)				
Physical activity	Yes	216 (20.18)	69 (32.1)	0.5352	0.38–0.73	14.7035	0.0001
	No	854 (79.82)	146 (67.9)				
Diet pattern	Adequate	793 (74.11)	168 (78.13)	0.8009	0.56–1.13	1.5399	0.2146
	Inadequate	277 (25.89)	47 (28.87)				
Routine meals	3 times	603 (56.35)	123 (57.2)	0.9658	0.71–1.29	0.0531	0.817
	Skipping meals	467 (43.65)	92 (42.8)				
Consumption of fast food	Yes	686 (64.11)	98 (45.6)	2.1328	1.58–2.86	25.84	0.0001
	No	384 (35.89)	117 (54.4)				
Sleep hours	=8 hours	135 (12.61)	67 (31.16)	0.3319	0.23–0.46	43.477	0.0001
	<8 hours	935 (87.39)	154 (68.84)				

**Table 5:** Association between behavioral risk factors and obesity.

Risk factors	Behavior	Total participants (*n*=1070) (%)	Obesity frequency (*n*=48) (%)	Odds ratio	95% CI	Chi-square value	*P* value
Smoking habit	Yes	538 (50.28)	27 (56.25)	0.7691	0.43–1.38	0.7835	0.376
	No	532 (49.72)	21 (43.75)				
Physical activity	Yes	216 (20.18)	4 (8.3)	2.7822	0.99–7.83	4.0838	0.0432
	No	854 (79.82)	44 (91.7)				
Diet pattern	Adequate	793 (74.11)	17 (35.41)	5.22	2.90–9.60	34.46	0.001
	Inadequate	277 (25.89)	31 (64.59)				
Routine meals	3 times	603 (56.35)	11 (22.91)	4.3432	2.19 –8.60	20.74	0.001
	Skipping meals	467 (43.65)	37 (77.09)				
Consumption of fast food	Yes	686 (64.11)	19 (39.58)	2.7267	1.50–4.92	11.86	0.0005
	No	384 (35.89)	29 (60.42)				
Sleep hours	=8 hours	135 (12.61)	7 (14.58)	0.8457	0.37–1.92	0.1602	0.688
	<8 hours	935 (87.39)	41 (85.42)				

## Data Availability

All data supporting this study are provided as supplementary information accompanying this paper in Excel format.

## Additional Points

*Contribution of the Study*. The findings of the present study are of inestimable worth to support advocacy of the modified risk indicators for the increasing magnitude of noncommunicable disease. This research work delivers the first and most comprehensive evidence linking behavioral risk factors with increased occurrence of noncommunicable diseases in the region. Therefore, it is important to address the impacts of these behavioral habits and health hazards. It is an essential requirement to provide suitable measures combined with stronger and sustainable plans to change these behaviors and their use. *Limitation of the Study*. The study has been conducted in the specific primary health centers of Asir region.

## References

[1] Ismail I., Venugopalan P., Sarada A., Binub K. (2016). Prevalence of non-communicable diseases risk factors among college students of Anjarakandy Integrated Campus, Kannur, Kerala, India. *Journal of Medical Society*.

[2] World Health Organization (2017). *Non-Communicable Disease Surveillance*.

[3] World Health Organization (2015). *Noncommunicable Diseases Country Profiles 2014*.

[4] Epping-Jordan J. E., Galea G., Tukuitonga C., Beaglehole R. (2005). Preventing chronic diseases: taking stepwise action. *The Lancet*.

[5] Alhyas L., McKay A., Balasanthiran A., Majeed A. (2011). Prevalences of overweight, obesity, hyperglycemia, hypertension, and dyslipidemia in the gulf: a systematic review. *JRSM Short Reports*.

[6] Amin T. T., Al Sultan A. I., Mostafa O. A., Darwish A. A., Al-Naboli M. R. (2014). Profile of non-communicable disease risk factors among employees at a Saudi university. *Asian Pacific Journal of Cancer Prevention*.

[7] Oommen A., Abraham V., George K., Jose V. (2016). Prevalence of risk factors for non-communicable diseases in rural & urban Tamil Nadu. *Indian Journal of Medical Research*.

[8] Yusuf N. (2014). Private and public healthcare in Saudi Arabia: future challenges. *International Journal of Business and Economic Development*.

[9] Central Department of Statistics and Information (CDSI). http://www.cdsi.gov.sa/english, 2017

[10] United Nations Development Programme (2010). *Human Development Report 2010. The Real Wealth of Nations: Pathways to Human Development*.

[11] Walston S., Al-Harbi Y., Al-Omar B. (2007). The changing face of healthcare in Saudi Arabia. *Annals of Saudi Medicine*.

[12] Alzeidan R., Rabiee F., Mandil A., Hersi A., Fayed A. (2016). Non-communicable disease risk factors among employees and their families of a Saudi university: an epidemiological study. *PLoS One*.

[13] Al-Hazzaa H. M., Alahmadi M. A., Al-Sobayel H. I., Abahussain N. A., Qahwaji D. M., Musaiger A. O. (2014). Patterns and determinants of physical activity among Saudi adolescents. *Journal of Physical Activity and Health*.

[14] Olayiwola K., Soyibo A., Atinmo T. (2003). Impact of globalization on food consumption, health, and nutrition in Nigeria. http://www.fao.org/tempref/docrep/fao/007/y5736e/y5736e01.pdf.

[15] Timlin M. T., Pereira M. A., Story M., Neumark-Sztainer D. (2008). Breakfast eating and weight change in a 5-year prospective analysis of adolescents: project EAT (eating among teens). *Pediatrics*.

[16] Affenito S. G., Thompson D. R., Barton B. A. (2005). Breakfast consumption by African-American and white adolescent girls correlates positively with calcium and fiber intake and negatively with body mass index. *Journal of the American Dietetic Association*.

[17] Dunn R. A., Sharkey J. R., Horel S. (2012). The effect of fast-food availability on fast-food consumption and obesity among rural residents: an analysis by race/ethnicity. *Economics and Human Biology*.

[18] Athens J. K., Duncan D. T., Elbel B. (2016). Proximity to fast-food outlets and supermarkets as predictors of fast-food dining frequency. *Journal of the Academy of Nutrition and Dietetics*.

